# Morphometric evaluation of wound healing in burns treated with Ulmo (*Eucryphia cordifolia*) honey alone and supplemented with ascorbic acid in guinea pig (*Cavia porcellus*)

**DOI:** 10.1186/s41038-016-0050-z

**Published:** 2016-10-03

**Authors:** Carolina Schencke, Adriana Vasconcellos, Cristian Sandoval, Paulina Torres, Francisca Acevedo, Mariano del Sol

**Affiliations:** 1 Doctoral Program in Morphological Sciences, Universidad de La Frontera, Temuco, Chile; 2 CONICYT-PCHA/National Doctorate Programme/2014-21141130, Santiago, Chile; 3 Facultad de Medicina, Universidad de La Frontera, Temuco, Chile; 4 CIMA Research Group, Facultad de Odontología, Universidad de La Frontera, Temuco, Chile; 5 CONICYT-PCHA/Doctorado Nacional/2015-21150991, Santiago, Chile; 6 Scientific and Technological Bioresource Nucleus, Universidad de La Frontera, Temuco, Chile; 7 Centro de Excelencia en Estudios Morfológicos y Quirúrgicos (CEMyQ), Universidad de La Frontera, Temuco, Chile; 8 Centro de Investigación en Ciencias Biomédicas, Universidad Autónoma de Chile, Temuco, Chile

**Keywords:** Honey, Ascorbic acid, Burn, Morphometry, Wound healing

## Abstract

**Background:**

In the context of the search for cost-efficient treatments, Ulmo (Eurcyphia cordifolia) honey is an excellent alternative for treating burn wounds and could have a profound medical, social, and economic impact. Ascorbic acid is an enzymatic co-factor necessary for the synthesis of collagen and the proliferation of fibroblasts and has been proposed as a coadjuvant to strengthen the healing effects of honey. The aim of this work was to evaluate by morphometric studies the healing wounds caused by burns treated with Ulmo honey alone and supplemented with ascorbic acid in guinea pig (*Cavia porcellus*).

**Methods:**

Fifteen guinea pigs were used and divided randomly into three groups: positive control (C+), experimental with unsupplemented honey (H), and experimental with supplemented honey (SH). A uniform deep burn covering 1 cm^2^ of the back skin was performed. The following indices were calculated for the morphometric study: superficial contraction index of the wound, deep contraction index of the wound, wound severity index, global healing index, global contraction index, and dermal proliferation area.

**Results:**

The superficial contraction index of the wound, global healing index, global contraction, and dermal proliferation area values of the experimental with supplemented honey group were higher than the other groups (*P* < 0.05).

**Conclusions:**

According to these results, the combination of honey with an antioxidant (ascorbic acid) promotes an appropriate action to support the healing effect. This study showed that by supplementing the Ulmo honey with ascorbic acid, the healing and contraction effects can be strengthened in burn wounds compared to unsupplemented honey. These results were proof of the synergy between honey and ascorbic acid in healing burn wounds.

## Background

A wound is defined as an interruption in the continuity of tissue, causing cell destruction, alteration of blood vessels, loss of blood components, and hypoxia. Wound healing (scar tissue formation) is a process involving three phases: inflammation, proliferation, and remodeling. It results from a series of interactions between cytokines, growth factors, blood components, and cell elements, which promote the production of components of the basal membrane, prevent dehydration, and increase inflammation and the formation of granulation tissue [1].

Burns are oxidative injuries, which increase the activity of free radicals in the damaged area, resulting in an increase in lipid peroxidation. According to clinical studies, early application of honey to burns has proven effective since it acts on free radicals, which validates its use on lesions of this kind [2, 3]. Furthermore, treatment with honey has presented significantly faster healing of burns than treatment with silver sulfadiazine, polyurethane film, and amniotic membrane [4, 5]. The anti-microbial, anti-inflammatory, and autolytic debridement activity of honey has also been validated [6–8].

Honey consists of water, sugars (especially glucose and fructose), antioxidants, amino acids, vitamins, minerals, glucose oxidase, and gluconic acid, which gives rise to honey’s acidity (pH 3.2 to 4.5). The medical properties of honey depend on its chemical composition, which varies principally as a function of the plant from which it is derived. This has led to the development of various products based on monofloral honeys, for example, the therapeutic Medihoney™ and Active Manuka Honey®, both made from the pollen of the Manuka tree (Europe). The establishment of a controlled designation of origin gives value-added honey. In Chile, Ulmo (*Eucryphia cordifolia*) honey has been shown to possess excellent bactericidal [9] and healing properties [10].

The stimulant effect of honey is due not only to its sugar content but to other constituents that act in synergy. Thus, the reduction in healing time may be the result of a double effect. On the one hand, prolonged inflammatory response is reduced by suppression of the production and propagation of inflammatory cells at the wound site; on the other, the production of pro-inflammatory cytokines is stimulated, resulting in a proliferation of fibroblasts and epithelial cells [11, 12].

The stress associated with a burn lesion increases the need for ascorbic acid, which is required for collagen synthesis, angiogenesis, and as an antioxidant [13, 14], as has been extensively studied in guinea pigs [15–17]. Horton [18] examined the effects of the treatment based on the administration of vitamins after burn trauma, showing the effects on the synthesis of cardiomyocytes and the secretion of pro-inflammatory cytokines. He also indicated that the combination of antioxidants (vitamins C, E, and A) could provide useful support to the healing effect.

Given the complexity of treating burns and the need for experimental studies offering similar or better alternatives to conventional treatments, the aim of this work was to carry out a histological and morphometric study of the effect of Ulmo honey supplemented with ascorbic acid on the healing and contraction of burn wounds and compare this effect with that of unsupplemented Ulmo honey.

## Methods

### Physical and chemical properties of honey

Samples of Ulmo honey were harvested on March 2014 from various apiaries in the Valdivian forest of southern Chile. The honeys were stored at 4 °C and homogenized with a stirrer before measurement. The Ulmo honey was sterilized by gamma irradiation at 25 kGy. The pollen was identified by melissopalynology using light microscopy analysis as per NCh2981 [19]. The levels of sporulating aerobic mesophilic microorganisms, anaerobic sulfite-reducing microorganisms, molds, and yeasts in irradiated Ulmo honey were determined by Association of Official Analytical Chemists (AOAC) methods [20]. Microbial counts were expressed as colony-forming units per gram (cfu/g) of honey. The mixture (Ulmoplus) was prepared using Ulmo honey supplemented with a solution of ascorbic acid. The physical and chemical properties of Ulmo honey and supplemented Ulmo honey were determined as follows: the viscosity was measured with a double cylinder digital high-speed viscometer (digital viscometer, model MRC, VIS-79 series; MRC, Israel) at 20±2 °C. To determine the pH, 5 g of homogenized honey was mixed with 20 mL of distilled water and the pH of the samples was measured using a digital pH meter. Samples of honey were analyzed for 5-hydroxymethylfurfural (HMF) content according to the spectrophotometric method [21]. Five grams of honey was dissolved in 25 mL of water, transferred quantitatively into a 50-mL volumetric flask; 0.5 mL of Carrez solution I and 0.5 mL of Carrez II were added and the volume was made up to 50 mL with water. The solution was filtered through paper, and the first 10 mL of the filtrate were rejected. Aliquots of 5 mL were put into two test tubes: one tube was added with 5 mL of distilled water (sample solution) and the second was added with 5 mL of sodium bisulfite solution 0.2 % (reference solution). The absorbance of the solutions at 284 and 336 nm was determined using a UV-visible spectrophotometer (Genesys 6, Thermo Scientific, USA). The quantitative HMF value was calculated according to the following equation:

$$ \frac{\mathrm{mg}\ \mathrm{H}\mathrm{M}\mathrm{F}}{100\ \mathrm{g}\ \mathrm{honey}}=\frac{\left[\left(\mathrm{Abs}\ 284\ \mathrm{nm}-\mathrm{Abs}\ 336\ \mathrm{nm}\right) \times 5\right]}{\mathrm{g}\ \mathrm{sample}} $$

Diastase activity was measured according to the harmonized method of the European Honey Commission. An insoluble blue dye cross-linked with the type of starch was used as the substrate. This was hydrolyzed by the enzyme, yielding blue, water-soluble fragments, determined photometrically by using a UV–Visible spectrophotometer (Genesys 6, Thermo Scientific, USA) at 660 nm. The absorbance of the solution was directly proportional to the diastatic activity of the sample. The diastase activity was calculated as its diastase number (DN). DN expresses units of diastase activity (Gothe unit). One unit is defined as the amount of enzyme that will convert 0.01 g of starch to the prescribed end point at 40 °C under test conditions [22].

The results of the physical and chemical properties of honey were analyzed statistically using the Student’s *t* test (SPSS, version 20.0). Differences between mean values were considered significant at *p* < 0.05.

### Wound model

**Fig. 1:**
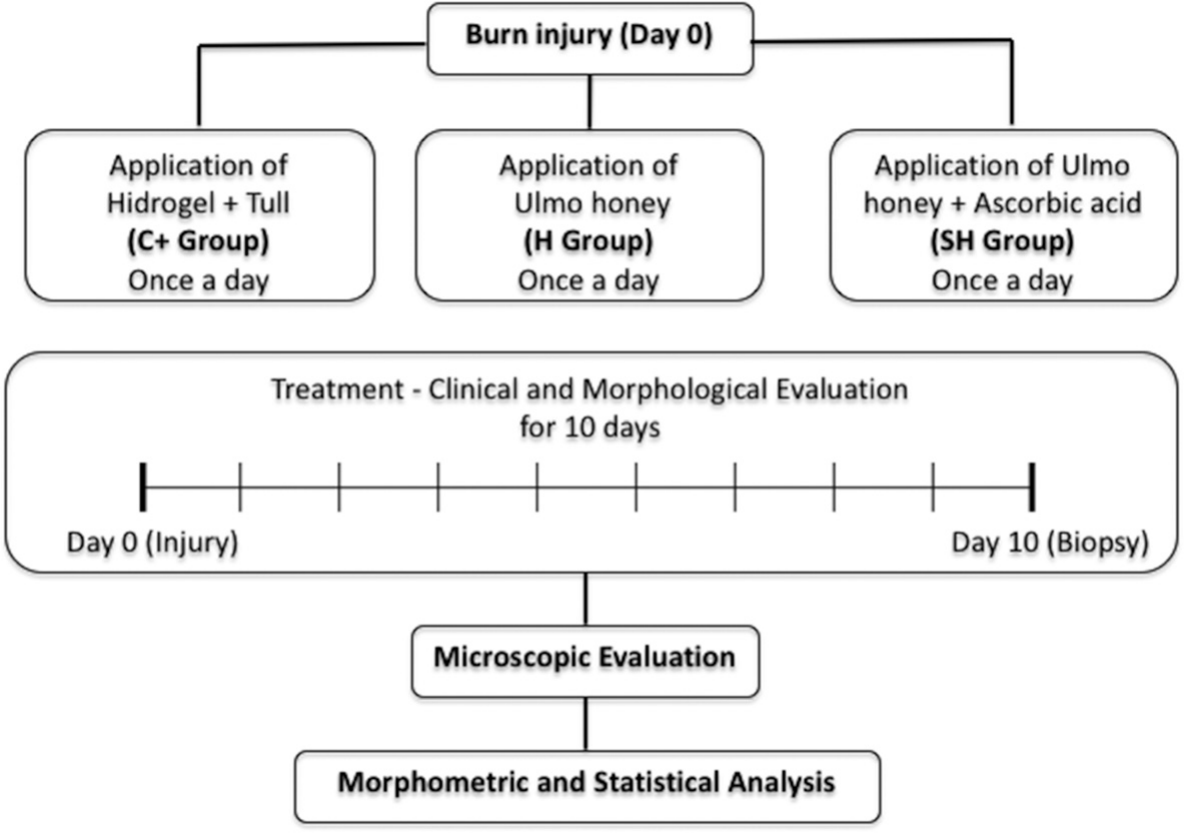
Working protocol for the analysis of the treatments and comparative study. The animals were divided at random into three groups: positive control (C+), unsupplemented Ulmo honey (H), and supplemented Ulmo honey (SH)

Guinea pigs were used as animal models because their metabolism is dependent on ascorbic acid. Fifteen healthy adult guinea pigs (*Cavia porcellus*) were used, of both sexes, average weight 450 g [23], fed on pellets supplemented with ascorbic acid and water ad libitum, under ambient conditions controlled for temperature (18–24 °C), ambient noise, and a cycle of 12 h light–darkness in the Centro de Excelencia en Estudios Morfológicos y Quirúrgicos (CEMyQ) at the Universidad de La Frontera, Chile. The animals were divided at random into three groups: positive control (C+), Ulmo honey only (H), and supplemented honey (SH) (Fig. 1). Two test guinea pigs were also used to obtain a biopsy of the healthy skin to assess normality. A uniform deep burn of the back skin was performed with a hot metal object (1000 ± 1 °C) during an exposure for 3 s. A biopsy of the burned area was extracted. The extracted region was a circular area of 1 cm^2^. Intraperitoneal anesthesia was applied with a mixture of ketamine (40 mg/kg), xylazine (5 mg/kg), and atropine (0.05 mg/kg). The burns were treated by the application of tepid physiological serum by syringe at a distance of 10 cm from the lesion; gauze impregnated with unsupplemented Ulmo honey was applied to the H group, Ulmo honey supplemented with ascorbic acid to the SH group, and hydrogel-tull to the C+ group. The animals were treated with this procedure, and the wounds were evaluated daily until biopsies were taken at day 10 post-injury. Day 10 post-treatment was selected because that day was representative of the proliferative phase of wound healing on research done previously [24]. The experiments were carried out in accordance with the Protocol for the Daily Supervision of Animals from the Guide to Bioethical Aspects of Animal Experiments [25]. The protocol for the experiment was approved by the Scientific Ethics Committee of the Universidad de La Frontera, Chile.

### Processing of biopsies and staining

The biopsies were washed in NaCl 0.9 %, fixed in buffered formalin (1.27 mol/L of formaldehyde in a phosphate 0.1 M buffer, pH 7.2) at 10 % for 48 h, dehydrated, and soaked in Paraplast Plus (Sigma-Aldrich Co., St. Louis, MO, USA). Once the blocks had been obtained, serial cuts were made in each block. Five cuts were taken at random (Microm HM 325 microtome) and stained with H&E for histopathological and morphometric analysis. Histopathological analysis was carried out in a Leica® DM 750 optical microscope, with a Leica® ICC50 HD camera. The images were projected in a LED LG® 55UB8300.

### Morphometric analysis

**Fig. 2:**
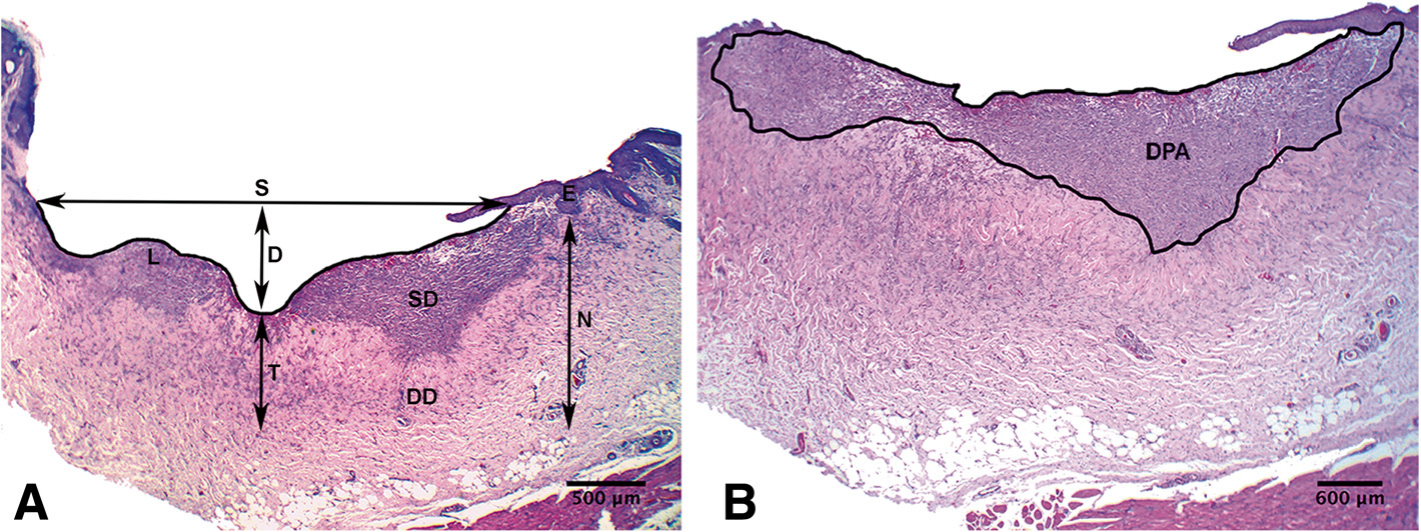
Measurement parameters for obtaining morphometric healing indices. **a** Photograph of a treated biopsy of unsupplemented Ulmo honey (H group) with the parameters used, known as lines *L*, *S*, *N*, *T*, and *D*. **b** Line drawn with freehand-line of the ImageJ Software to mark the dermal proliferation area (DPA). *SD* superficial dermis, *DD* deep dermis

Five slides per individual were observed, totaling 25 cuts per group. The slides were observed with a Motic® SMZ–171 stereoscopic microscope and photographed with a Moticam® 580 camera. Five morphometric measurements were taken [26], known as lines L, S, N, T, and D (Fig. 2a), where

- *L* is the length of the re-epithelization zone, i.e., the length of the tissue (new or exposed dermis) between the borders of the wound.
- *S* is the distance between the borders of the wound, following the straight line of the epidermis.
- *D* is the depth of the wound, from the line of the epidermis (line S) to the first layer of connective tissue at the deepest point of the wound.
- *T* is the thickness of the connective tissue (residual or new dermis) in the center of the wound, from the deepest point of the wound in the muscle.
- *N* is the thickness of the natural dermis on both sides of the wound, from the muscle to the epidermis. The classic way of calculating *N* is *N* = *D* + *T*..

These allowed the following morphometric indices to be calculated:

- Superficial contraction index (SCI) of the wound
  $$ \mathrm{S}\mathrm{C}\mathrm{I}=\frac{\left(L-S\right)}{L} $$
- Deep contraction index (DCI)
  $$ \mathrm{D}\mathrm{C}\mathrm{I}=\frac{\left(N-D\right)}{N} $$
- Wound severity index (WSI)
  $$ \mathrm{W}\mathrm{S}\mathrm{I}=\frac{\left(N-T\right)}{N} $$
- Global healing Index (GHI)
  $$ \mathrm{G}\mathrm{H}\mathrm{I}=\mathrm{S}\mathrm{C}\mathrm{I}+\mathrm{D}\mathrm{C}\mathrm{I}-\mathrm{W}\mathrm{S}\mathrm{I} $$
- Global contraction index (GCI)

$$ \mathrm{G}\mathrm{C}\mathrm{I}=\mathrm{S}\mathrm{C}\mathrm{I}+\mathrm{D}\mathrm{C}\mathrm{I} $$

The dermal proliferation area (DPA) was calculated using the Freehand-line tool from ImageJ® Software (Fig. 2b). The parameters were measured with ImageJ® Software. The indices used show greater healing and wound contraction as the value approaches 1. In the WSI, the severity decreases as the value approaches 0.

### Debridement analysis

Debridement generated by the different treatments was clinically evaluated.

### Statistical analysis

The statistical analysis was performed with IBM SPSS Statistic 21© software, and the assumptions were verified with the one-sample Kolmogorov–Smirnov test (data normality test) and Levene’s test (homogeneity test of variance). For the analysis of the differences between groups, a one-way analysis of variance (ANOVA) and Tukey’s post hoc HSD or Dunnett’s T3 tests were used to analyze the differences between groups. The *P* values were considered significant when less than 0.05 (*) and very significant when less than 0.01 (**).

## Results

### Pollen identification of Ulmo honey

The identification of Ulmo honey pollen indicated that the sample is composed mainly of *Eucryphia cordifolia* and other species in insignificant amounts by comparison (Table 1). The sample analyzed was therefore classified as a monofloral honey since more than 45 % of the pollen content originates from a single plant species [27]. The results for all the microbiological parameters analyzed were below the detection limit (<10 cfu/g): sporulating aerobic mesophilic microorganisms, anaerobic sulfite-reducing microorganisms, and the fungi and yeast count.

**Table 1:** Pollen identification of Ulmo honey

Scientific name	Pollen in sample (%)
*Eucryphia cordifolia*	88.03
*Lotus uliginosus*	6.18
*Luma apiculata*	2.68
*Caldcluvia paniculata*	0.67
*Cissus striata*	0.67
*Trifolium pratense*	0.53
*Trifolium repens*	0.53
*Leontodon taraxacoides*	0.40
*Plantago lanceolata*	0.26

### Physical and chemical properties of H and SH

The physical and chemical properties, e.g., viscosity, pH, HMF, and diastase activity, were determined for irradiated Ulmo honey (H) and irradiated Ulmo honey supplemented with ascorbic acid (SH). The results are shown in Table 2.

**Table 2:** Physical and chemical parameters of H and SH

Parameter	H	SH
Color (Pfund scale, mm)	73.955 (Light amber)	67.517 (Light amber)
pH*	3.475 ± 0.030	3.370 ± 0.050
Viscosity (mPa*s)*	28,005 ± 552.56	12,721 ± 134.57
Moisture (mg/100 g)	18.133 ± 0.155	16.670 ± 3.820
Total solids (mg/100 g)	81.867 ± 0.115	81.600 ± 3.686
Ash (mg/100 g)*	0.282 ± 0.032	0.032 ± 0.003
Hydroxymethylfurfural (HMF) (mg/Kg)*	2.76 ± 0.001	1.68 ± 0.008
Reducing sugars (g/100 g)*	82.868 ± 0.663	89.540 ± 0.786
Total sugars (g/100 g)	97.419 ± 1.018	97.885 ± 1.271
Diastase activity (Gothe scale)	6.82	4.93
Proteins (mg/g dm)	12.644 ± 0.289	12.839 ± 1.566

*H* unsupplemented Ulmo honey, *SH* supplemented Ulmo honey

*Significantly different according to the Student’s *t* test (*p* < 0.05)

Results showed that there were statistically significant differences for hydroxymethylfurfural, reducing sugars and ash contents, and pH and viscosity values, between H and SH.

### Histopathology

The biopsies of healthy skin presented a thin epidermis with a thick corneal layer. The superficial papillary dermis consisted of lax connective tissue in which pilous follicles and sebaceous glands were observed; the deep dermis presented abundant, randomly distributed collagen bundles. In the test animal for evaluation of the degree of the lesion, signs of inflammation and reddening were observed immediately. The biopsy showed deep skin damage. There was no evidence of any adverse reaction to the honey used (e.g., edema, marked accumulation of exudates).

**Fig. 3:**
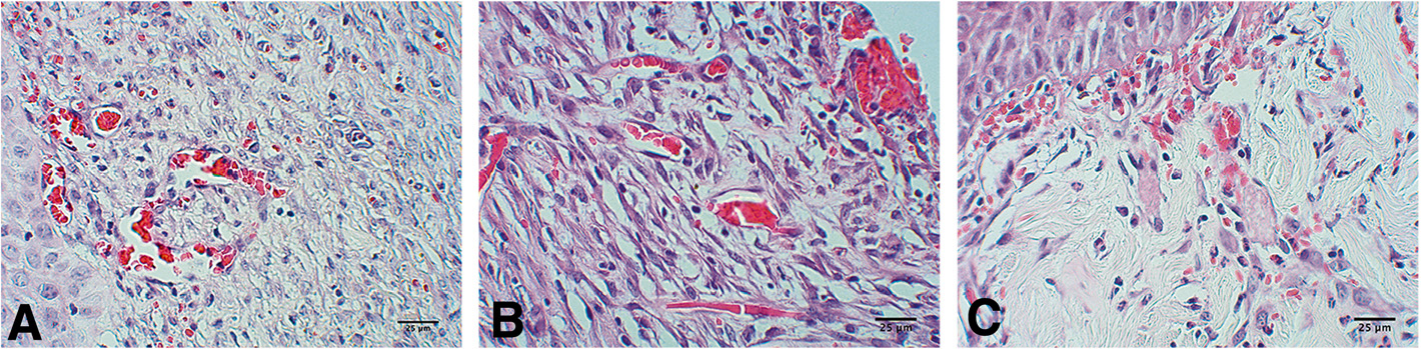
H&E staining for each study group: positive control (C+), unsupplemented Ulmo honey (H), and supplemented Ulmo honey (SH). **a** Biopsy from the C+ group. Neoformation of blood vessels is observed only in the superficial dermis. **b** The H group. Neoformation of blood vessels is observed in all the scar tissue of the dermis, especially the superficial dermis where there is as yet no epidermal development. **c** The SH group. Numerous blood vessels are observed in all the scar tissue of the dermis, and there are abundant small capillaries in the superficial dermis

In the C+ group, the eschar was shed spontaneously on days 8–9 post-injury. It presented a diameter of 6 ± 1 mm, with a reduction of 40 % on day 10 of treatment. The biopsies presented an advanced proliferative phase with fibroblast reaction. Epidermal regeneration was observed in 40 % of the biopsies at the differentiation stage. In the superficial dermis, neoformation of blood vessels was observed as well as connective tissue with abundant collagen fibers, regularly ordered and differentiated from the hypodermis, where they formed thick bundles (Fig. 3a).

In the H group, the eschar was shed spontaneously at days 6–7 post-injury, exposing a wound of 5 ± 1 mm diameter, no bleeding, with granular tissue, and no macroscopic evidence of epithelization. The diameter diminished to 50 % by day 10 post-injury. No epidermal regeneration was observed. In the scar tissue of the dermis, an initial proliferative stage was observed with abundant cellularity and active fibroblasts and neoformation of blood vessels mainly in the superficial dermis (Fig. 3b).

The SH group shed the eschar spontaneously on day 6 post-injury, exposing a wound of 5 ± 1 mm diameter. The diameter dropped to approximately 50 % by day 10 post-injury. The epidermis regenerated in around 60 % of the biopsies, with no evidence of a corneal layer. The remaining layers were differentiated: the basal layer was well developed, with a differentiated spinous layer and indications of a granular layer. Numerous blood vessels and small capillaries were observed in the superficial dermis (Fig. 3c); the scar tissue zone presented a fibroblastic reaction, proliferation of collagen fibers, dense connective tissue, and thin collagen fibers in an advanced proliferative stage. No pilous follicles or sebaceous glands were observed.

### Morphometric indices

**Fig. 4:**
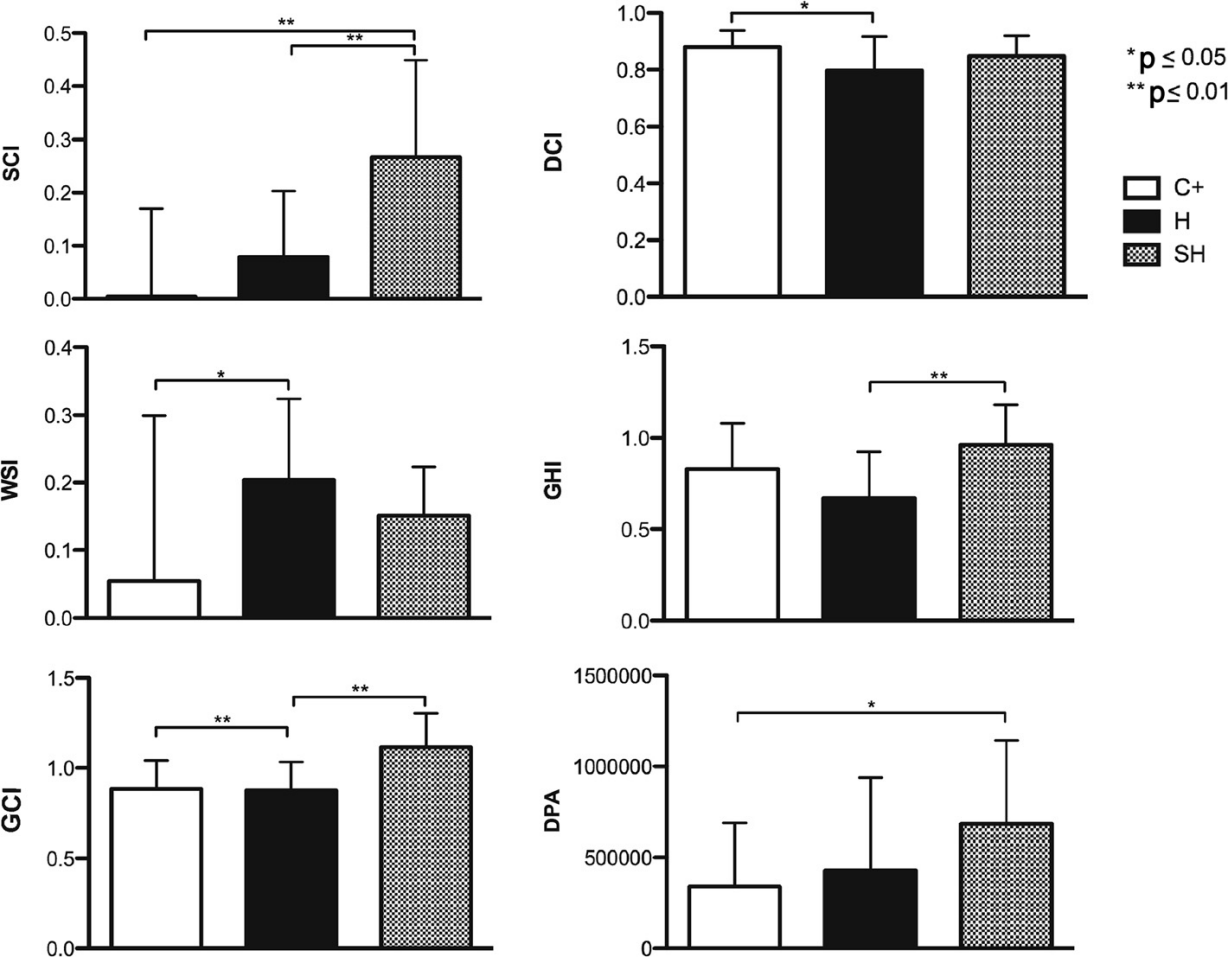
Analysis of the morphometric measurements carried out on the biopsies of the treated burns, taken on day 10 post-treatment. The morphometric healing indices (*SCI*, *DCI*, *WSI*, *GHI*, *GCI*, and *DPA*) obtained from the groups: positive control (C+), unsupplemented Ulmo honey (H), and supplemented Ulmo honey (SH)

The ANOVA of the morphometric measurements carried out on the biopsies of the treated burns showed that for all the indices, there was at least one group which differed from the others (*P* < 0.05). The Tukey’s post hoc HSD test for the SCI of the wound showed that statistically significant differences exist between the C+ group and the experimental groups (H and SH) (*P* = 0.000), whereas for the DCI and the WSI, statistically significant differences were only found between the C+ group and the H group (*P* < 0.025). For the GHI and the GCI, differences were observed between the H and SH groups (*P* < 0.025). Finally, for the DPA, statistically significant differences were observed between the C+ group and the SH group (*P* = 0.021) (Fig. 4).

### Debridement analysis

- *SH group*: The eschar fell off spontaneously at day 6 post-injury, with a 50 % reduction in its diameter at day 10 post-injury.
- *H group*: The eschar fell off spontaneously between 6 and 7 days post-injury, with a 50 % reduction in its diameter at day 10 post-injury.
- *C+ group*: The eschar fell off spontaneously between 8 and 9 days post-injury, with a 15 % reduction at day 10 of treatment.

## Discussion

The intrinsic properties of honey affect the growth and survival of many species of the microorganism. Among these properties are low pH and high sugar content. It has been reported that low pH values inhibit the presence and growth of microorganisms. Honeys are acidic due to the presence of organic acids that contribute to their flavor and stability against microbial spoilage. The pH values obtained for the H and SH groups were lower than the results reported for Moroccan honeys (3.55 to 4.28), Indian honeys (3.7 to 4.4), Algerian honeys (3.70 to 4.00), and Malaysian honeys (3.83 to 4.10) [28–31]. If honey is sterilized by gamma irradiation, it is to be expected that a small, restricted variety of microbes will be found in the honey [32]. The results obtained are consistent with the above and agree with the findings of Finola et al. [33]. They are much lower than those reported by Iurlina and Fritz [34], where the numbers of aerobic mesophilic bacteria, molds, and yeasts were less than 103 cfu/g for all samples analyzed. Both Ulmo honey and supplemented Ulmo honey could be used as potential antibacterial agents.

HMF content is widely known to be an indicator of honey freshness and high temperature processing. Both samples (Ulmo honey and supplemented Ulmo honey) presented an HMF level lower than the limit (40 mg/kg). These results suggested that the samples were fresh and had not been heat processed.

Wound healing involves the substitution of damaged tissues. Burn wounds suffer tissue destruction, which heals by the synthesis of fibrous tissue and tissue contraction. The latter implies great mechanical and physiological effort responsible for tissue retraction, depending on collagen synthesis and compaction of the granulation tissue. This supports the concept that the wound contraction mechanism occurs through locomotion rather than cell contraction forces and that the collagen is compacted by fibroblasts and not myofibroblasts [35]. Human keratinocytes, fibroblasts, and endothelial cell responses are positively affected in the presence of honey; thus, honey may accelerate re-epithelization and wound closure [36]. According to Khoo et al. [37], Tualang honey produced better wound contraction results in vivo than conventional medicine. In our study, the value of GCI in the H group was high (0.875 ± 0.158) and was similar to the C+ (0.883 ± 0.159), whereas it was much higher in the SH group (1.115 ± 0.188). Osuagwu et al. [38] stated that the contraction force in the wound is located in the granulation tissue, describing greater formation of granular tissue in wounds treated with honey than in the control group. We found that the DPA was better in the H group (426,739.7 ± 509) than in the C+ group (339,660.8 ± 350). The area was larger than both of these in the SH group (684,774.359 ± 459), in which the healing action of honey was strengthened. The GHI index presented higher values in the SH group (0.963 ± 0.22) than in the C+ (0.829 ± 0.253) and H groups (0.671 ± 0.252). The large difference between the values for the groups treated with supplemented and unsupplemented honey is striking; the effect of Ulmo honey is seen to be boosted by supplementing with ascorbic acid.

Honey may be considered an effective option for autolytic debridement [39]. It induces activation of the cells of the immune system, promoting wound debridement and so accelerating the repair process. The debriding action generated by the different treatments was evaluated clinically, observing an autolytic action in all groups. It was determined that the earlier the eschar came off, the faster the debriding action. Thus, the H and SH groups in the experiment presented excellent debridement, higher than the C+ group.

Cost-effectiveness is an important factor in treatment selection. According to clinical study by Moghazy et al., treatment with honey has shown a short healing interval, with a mean of 2.3 ± 0.94 weeks, while the duration with other treatments is considerably longer (17.7 weeks with conventional dressings, 15.7 with hyperbaric oxygen, and 6–8 weeks with laser therapy) [40]. The high index of wound contraction and healing obtained with the proposed treatment indicates that treatment with honey supplemented with ascorbic acid would be shorter than with honey alone or conventional dressings (synthetic hydrogel-tull). Our conclusions agree with Adewumi and Ogunjinmi [41], which given its cost-effectiveness, the incorporation of this alternative into health plans for burns and chronic wounds is indispensable, particularly in rural areas.

Guinea pigs were used as animal models because, unlike other mammals, their metabolism is dependent on ascorbic acid. The guinea pig is also an excellent model for healing studies since its skin thickness remains constant once its body weight exceeds 450 g [23].

## Conclusions

Honey supplemented with ascorbic acid may be an ideal substance for use in treatment as it is easy to apply and remove. Its low cost and absence of risk of antimicrobial resistance are the main arguments in favor of the application of this product for treating wounds compared to unsupplemented honey or the positive control. It was confirmed that the treatment in this study achieved effective, rapid, good quality healing. It was shown that supplementing the Ulmo honey with ascorbic acid boosted the healing and contraction effects on burn wounds compared to unsupplemented honey. These results are proof of the synergy between honey and ascorbic acid in healing burn wounds.

## Abbreviations

C+, positive control; DCI, deep contraction index of the wound; DN, diastase number; DPA, dermal proliferation area; GCI, global contraction index; GHI, global healing index; H, experimental group with unsupplemented honey; HMF, 5-hydroxymethylfurfural; SCI, superficial contraction index of the wound; SH, experimental group with supplemented honey; WSI, wound severity index.
